# FAM3C Regulates Glioma Cell Proliferation, Invasion, Apoptosis, and Epithelial Mesenchymal Transition via the Notch Pathway

**DOI:** 10.1002/cam4.70412

**Published:** 2024-12-04

**Authors:** Xiaochao Xia, Zihao Wang, Lvmeng Song, Yinchuan Cheng, Ping Xiong, Shun Li

**Affiliations:** ^1^ Departments of Neurosurgery Affiliated Hospital of North Sichuan Medical College Nanchong China

**Keywords:** epithelial mesenchymal transition, FAM3C, glioma, Notch pathway, therapeutic targets

## Abstract

**Background:**

Previous studies have implicated the involvement of FAM3C in cancerous development and progression. Herein, we aimed to further investigate the oncological mechanism of FAM3C, specifically in glioma.

**Methods:**

We utilized open‐source bioinformatics tools and platforms to analyze the transcriptional expression levels, prognosis, and correlation with clinical variables of FAM3C in gliomas, and subsequently, to hypothesize its potential molecular functions and possibly associated signaling pathways. Following this, glioma tissues were obtained from resected specimens of patients to assess the expression of FAM3C and molecular markers related to epithelial–mesenchymal transition (EMT) and Notch signaling pathways. Furthermore, glioma cell lines were subjected to treatments including FAM3C siRNA knockdown, lentiviral overexpression, and Notch signaling pathway blockade, enabling the investigation of molecular functions of FAM3C in vitro, particularly in EMT and Notch signaling pathways, as well as its effects on cancer cell proliferation, cell cycle progression, apoptosis, and invasion, using assays such as MMT cell proliferation assay, transwell migration, and flow cytometry analysis. Finally, a mouse subcutaneous xenograft model was established to explore the integrative function of FAM3C in glioma growth in vivo.

**Results:**

The expression level of FAM3C correlated with the progression of glioma grade and served as a prognostic indicator for poor patient outcomes. Subsequent experiments conducted on glioma cell lines, tumor tissues, and mouse models reinforced the close association of FAM3C with processes including glioma cell proliferation, cell cycle progression, apoptosis, and invasion. Additionally, it was observed that FAM3C is involved in the regulation of the Notch signaling pathway.

**Conclusions:**

FAM3C emerges as a potential candidate for clinical detection and prognostic biomarker application. Its regulatory role in glioma cell proliferation, cell cycle progression, and modulation of epithelial–mesenchymal transition‐driven invasion and migration via the Notch signaling pathway implies its potential to unveil novel therapeutic targets for glioma treatment.

## 1 Introduction

Gliomas, particularly glioblastoma (GBM), stand as the predominant primary malignancy within the human central nervous system [1]. Currently, the standard treatment modalities for this pathology have primarily relied on surgical resection combined with postoperative radiotherapy, with or without temozolomide. Nonetheless, the clinical challenges of rapid invasion, metastasis, high recurrence rate, and inherent chemo‐ and radio‐resistance have contributed significantly to elevated global mortality rates. Studies indicated that the median survival period for glioblastoma patients was merely 14.6 months [2, 3]. Hence, there exists a critical imperative to delve into the mechanisms underlying glioma development while identifying pertinent diagnostic markers and therapeutic targets.

The FAM3 gene family (family with sequence similarity of three member genes), consisting of FAM3A, FAM3B, FAM3C, and FAM3D, holds significant regulatory roles in the processes of angiogenesis and glucose and lipid metabolism, and none of them possesses a four‐helix structure. FAM3C adopts a β‐β‐α‐folded structure, capable of existing in both monomeric and covalent dimeric forms [4, 5, 6]. As a small secreted cytokine protein, FAM3C exhibits ubiquitous expression in human tissues and participates in various biological processes such as embryonic development, gluconeogenesis regulation, and carcinogenesis. Mounting evidence underscores FAM3C's overexpression in diverse human cancers, strongly linked to tumor initiation, invasion, metastasis, and poor survival. Its putative oncological mechanism involves promoting epithelial–mesenchymal transition (EMT) and enhances stem cell self‐renewal in tumor progression through the regulation of key molecules and signaling pathways [7, 8, 9]. Consequently, targeting FAM3C functions emerges as a promising therapeutic strategy for FAM3C‐driven cancers.

Clinical data showed that glioma patients with FAM3C amplification had shorter disease/progression‐free intervals and overall survival compared to those without such amplification. While insufficient data preclude a direct association between FAM3C alteration and glioma patients' prognosis, the potential oncological role of FAM3C in gliomas has been implicated. Herein, our study investigated whether FAM3C contributes to glioma cell proliferation, invasion, apoptosis, and EMT by mediating the Notch signaling pathway, thus offering novel avenues for clinical diagnosis, prognostic assessment, and potential targeted therapy in gliomas.

## 2 Materials and Methods

### 2.1 Bioinformatics Analysis

We employed Gene Expression Profiling Interactive Analysis (GEPIA, http://gepia.cancer‐pku.cn/index.html) and the Chinese Glioma Genome Atlas (CGGA, http://www.cgga.org.cn/) to analyze FAM3C expression and its prognostic correlation in gliomas. Utilizing these resources, we accessed RNA‐seq and the corresponding clinical information from the CGGA database. Subsequently, the acquired data underwent preprocessing using R software (v5.1) and the Limma software package. This preprocessing involved steps such as data cleaning and normalization, enabling both unifactorial and multifactorial prognostic survival analyses to be conducted.

### 2.2 Tissue Samples

Between February 2022 and February 2023, we collected a total of 42 tumor tissue specimens from glioma patients. Among these, 10 specimens were classified as normal brain tissue, 9 as grade II, 11 as grade III, and 12 as grade IV. Specimens were promptly gathered following tumor resection and preserved in liquid nitrogen to prevent RNA from degradation. Notably, none of all patients had undergone radiotherapy or other treatments prior to surgery. Before tissue collection, patients or their representatives were duly informed and consented by signing a written consent form. The study adhered strictly to the principles outlined in the Declaration of Helsinki, and all experimental protocols were reviewed and approved by the Ethics Committee.

### 2.3 Cell Culture

HA1800, U87MG, and U251 cells were purchased from Keycell Biotechnology (Wuhan) Co. Ltd. Cells were cultured at 37°C in a 5% CO_2_‐humidified incubator. The medium used was Dulbecco's modified Eagle's medium with 10% fetal bovine serum (FBS) (Gibco, USA) and 1% penicillin/streptomycin (Corning, USA). Cell passaging was carried out in 1:3, and the solution was changed every 2–3 days, and the next passaging was carried out when the cell growth fused to 70%–80%. Only cells in the logarithmic growth phase were selected for further experiments.

### 2.4 Cell Treatment and Grouping

To establish cell models exhibiting either low or high expression levels of FAM3C, siRNA (siFAM3C, sense, CGAUGAUGGAGCAACCAAATT; antisense, UUUGGUUGCUCCAUCAUCGTT)/plasmid constructs targeting FAM3C (pLVX‐siRNA2‐Puro‐hFAM3C, VP051‐CMV‐MCS‐EF1‐zsgreen‐T2A‐puro‐hFAM3C) with chemical modification, along with 10 μL of negative control products, were transfected into U87 and U251 cells at 30%–50% confluence. These cells were cultured in six‐well plates using a reagent system in accordance with the product specifications. U87 and U251 cells in logarithmic growth phase, displaying robust growth, were selected for experimentation. The cells were then categorized into the following grouping: (1) U87/U251+NC, (2) U87/U251+FAM3C‐siRNA, (3) U87/U251+OE‐NC, (4) U87/U251+OE‐FAM3C, and (5) U87/U251+OE‐ FAM3C+DAPT (as a γ‐secretase inhibitor to block the Notch pathway, MCE, China). A viral titer of 1 × 10^8^ TU/mL was employed, and based on the MOI = 10 value, 20 μL of viral solution was added to each well. The cells were then incubated at 37°C in an incubator, and subsequent assay was conducted after 48 h of infection. The drug treatment protocol for DAPT was administered at a concentration of 2 μM and maintained for 48 h. The lentivirus‐based vectors for FAM3C overexpression and RNAi‐mediated knockdown of FAM3C were obtained from Gene Pharma (Gene Pharma Co. Ltd. China).

### 2.5 Real‐Time Quantitative PCR (RT‐qPCR)

Total RNA extraction was carried out using Trizol reagent (Ambion, USA), followed by reverse transcription of cDNA from the extracted total RNA using RT SuperMix (Vazyme, China). Real‐time quantitative PCR was performed using 2*Q3 SYBR qPCR Master Mix (Tolobio, China) and a Real‐Time Fluorescent Quantitative PCR System (ABI, USA), following a thermocycling profile comprising an initial denaturation step at 95°C for 3 min, followed by 40 cycles of denaturation at 95°C for 10 s, annealing at 60°C for 30 s, and extension at 72°C for 35 s. Data analysis was conducted using the DDCT method, with β‐actin serving as an internal control. The specific primer sequences utilized are outlined below:

- β‐actin forward: CCCTGGAGAAGAGCTACGAG.
- Reverse: CGTACAGGTCTTTGCGGATG.
- FAM3C Forward: TGCTGCAAAGTTGGGTGGTAG.
- Reverse: TCAGGGGCAAGCTTTTGAGAT.

### 2.6 Western Blot

Total proteins were extracted using the RIPA lysis extraction buffer solution reagent kit (Servicebio, China) and resolved by 10% SDS‐PAGE. Subsequently, the separated proteins were transferred onto a PVDF membrane (Millipore, USA). Visualization of the results was achieved using goat anti‐mouse IgG(H + L)‐HRP (Proteintech Group, USA) and goat anti‐rabbit IgG(H + L)‐HRP (Beyotime Biotechnology, China). The antibodies utilized in this study were as follows: Mouse monoclonal antibody β‐actin (Affinity, China), rabbit poly‐anti‐FAM3C (Proteintech Group, USA), rabbit poly‐anti‐E‐cadherin (Proteintech Group, USA), rabbit monoclonal antibody snail (CST, USA), rabbit poly‐anti‐twist (Affinity), rabbit multi‐anti‐HES1 (Affinity, China), rabbit multi‐anti‐HES5 (Affinity, China), and rabbit multi‐anti‐HEY1 (Proteintech Group, USA).

### 2.7 MTT Assay

U87 and U251 cells in logarithmic growth phase, exhibiting robust growth status, were harvested and seeded into 96‐well cell culture plates at a density of 5 × 10^3^ cells per well. Subsequently, 10 μL of MTT solution (MCE, China) was added to each well, followed by incubation at 37°C for 1 h. After removal of the medium, 150 μL of DMSO was added to each well and shaken for 10 min to solubilize the formazan crystals. The absorbance of each well was then measured at OD of 570 nm using an enzyme‐linked immunosorbent assay plate reader.

### 2.8 Transwell Method

In a 24‐well plate, 800 μL of 10% FBS DMEM (with double antibody) was added and placed into a precooled transwell (Falcon, USA) chamber at 4°C. Subsequently, 100 μL of Matrigel (Corning, USA) with a final concentration of 0.5 mg/mL was added vertically to the center of the bottom of the upper chamber of the transwell. The plate was then warmed at 37°C to allow the Marigel to dry into a gel. Once the Matrigel had solidified, 200 μL of each cell suspension was carefully added to the upper chamber of the transwell. The transwell assembly was then incubated at 37°C in a 5% CO_2_ incubator. Following incubation, the transwell was removed, and the chamber was gently washed once with PBS. The cells were fixed with 70% ice‐ethanol solution for 1 h, followed by staining with 0.5% crystal violet staining solution (Servicebio, China) at room temperature for 20 min. After staining, the cells were rinsed with PBS, and any nonmigrated cells on one side of the upper chamber were wiped off using a clean cotton ball. Finally, the migrated cells were examined, counted, and imaged using a digital microscope (200× magnification, five pictures per group).

### 2.9 Flow Cytometry Assay

After processing the cells for the required duration, trypsin digestion was performed to harvest the cells, followed by two washes with PBS and centrifugation at 1200 rpm for 5 min. The AnnexinV‐APC/7‐AAD Apoptosis Detection Kit instructions (Keygen Biotech Co. Ltd. China) were followed accordingly: 500 μL of Binding Bbuffer was added to resuspend the cells, followed by the addition of 5 μL of AnnexinV‐APC and thorough mixing; then, 5 μL of 7‐AAD was added. After resuspension in 500 μL of Binding Buffer, another 5 μL of AnnexinV‐APC and 7‐AAD were added, mixed thoroughly, and incubated for 5–15 min at room temperature in the absence of light. Simultaneously, a negative control was established using normal cells without AnnexinV‐APC and 7‐AAD. Subsequently, apoptosis was detected using a flow cytometer (BECKMAN, USA).

### 2.10 In Vivo Analysis

All animal experiments were conducted in accordance with the guidelines set forth by the Ethics Committee. U251MG cells were transfected with lentiviruses encoding si‐FAM3C and OE‐FAM3C to achieve stable expression. In the OE‐FAM3C + DAPT group, 15 mg/kg of DAPT was administered via intraperitoneal injection every 3 days in the OE‐FAM3C mouse model. These cells were then injected subcutaneously at 200 μL into the left side of the nude mice. Daily monitoring of the mice's health and tumor growth was performed, with tumor dimensions measured every 3 days using Vernier calipers. Tumor volume (*V*) was calculated using the formula *V* = *a* * *b* ^2^/2, where “*a*” represents the longest diameter and “*b*” represents the shortest diameter of the tumor. After 28 days, the mice were euthanized, and the tumors were excised. Tumors were arranged systematically on a monochrome background and photographed alongside a ruler for size reference. A portion of tumor tissues was fixed with 4% paraformaldehyde, while another portion was frozen and stored for subsequent analyses.

### 2.11 Histological Analysis

Hematoxylin and eosin (H&E) staining, along with immunohistochemical (IHC) staining, was performed following established protocols. Mouse tumor samples were fixed overnight in 4% paraformaldehyde in PBS, paraffin‐embedded (SINOPHARM, China), and sectioned into 5 μm thick slices. IHC analysis was conducted using specific antibodies to evaluate the corresponding protein levels and distribution. Stained sections were examined and photographed under a light microscope. The primary antibodies used in this study are shown in Table 1.

**TABLE 1:** Primary antibodies used in the immunohistochemistry (IHC) experiments.

Antibodies	Supplier	Catalog number	Dilution
FAM3C	Abcam	Ab72182	1:100
Ki67	Abcam	Ab17666	1:100
E‐cad	Proteintech	20874‐1‐AP	1:100
Snail	Abcam	Ab224731	1:100
Twist	Proteintech	25465‐1‐AP	1:100
HES1	Abcam	Ab108937	1:100
HES5	Abcam	Ab194111	1:100
HEY1	Bioss	Bs‐16500	1:100

### 2.12 Statistical Analysis

Data were presented as mean ± SEM, with all experiments repeated at least three times for robustness. Differences in categorical variables were analyzed using the Chi‐squared test. Comparison between different groups of data was performed using one‐way ANOVA and Student's *t* tests. Statistical analyses were carried out using the software GraphPad Prism 9.5.1 (GraphPad, La Jolla, CA, USA) and SPSS statistical software, version 27.0.1 (IBM Corp, Armonk, NY, USA). A *p*‐value less than 0.05 was considered statistically significant.

## 3 Results

### 3.1 FAM3C Is Highly Expressed in Gliomas and Affects Patients' Prognostic Survival

**FIGURE 1:**
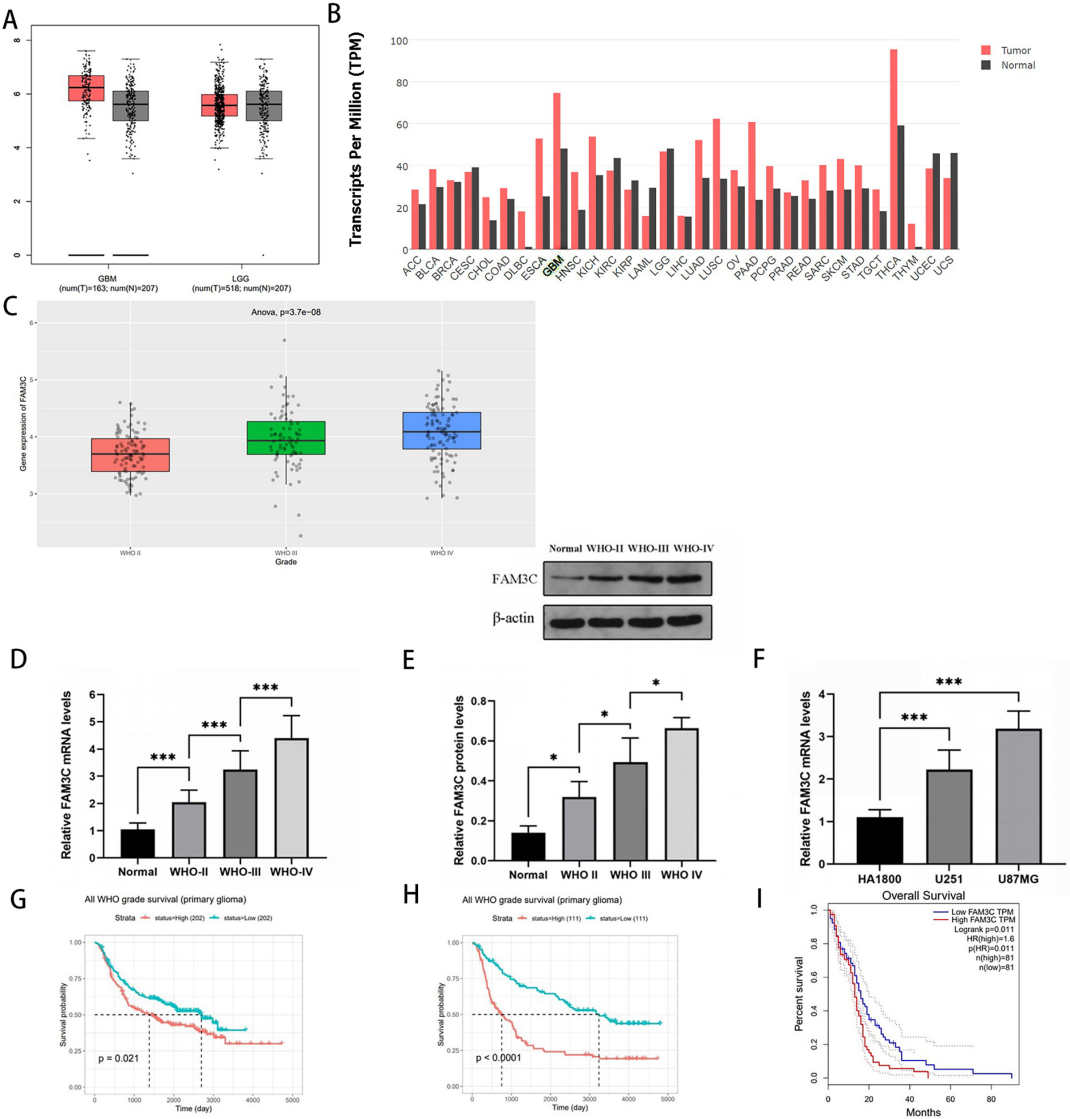
FAM3C is highly expressed in gliomas and affects patients' prognostic survival. (A, B) FAM3C expression in gliomas as well as other tumors in the GEPIA online database. (C) Relationship between FAM3C expression levels and WHO grading of glioma pathology in the CGGA database. (D, E) Expression levels of FAM3C in different WHO‐graded glioma tissues and normal brain samples by RT‐qPCR and western blot. (F) Expression levels of FAM3C in glioma cell lines (U251, U87MG) and normal cell line (HA1800) were detected by RT‐qPCR. (G–I) Effects of genetic alterations of FAM3C on overall survival in gliomas analyzed in the CGGA325, CGGA693, GEPIA database. *p < 0.05; ***p < 0.001.

Initial analysis revealed a notable upregulation of FAM3C expression in glioblastoma compared to normal brain tissues, as evidenced by RNA‐seq data sourced from GTEx and TCGA datasets within the GEPIA database (Figure 1A,B). Moreover, this elevation was found to be significantly higher in glioblastoma compared to various other tumor types. Subsequent examination of the CGGA database further corroborated these findings, demonstrating a positive correlation between FAM3C expression levels and the pathologic WHO classification of gliomas (Figure 1C). Utilizing qRT‐PCR and western blotting, we assessed FAM3C expression levels in glioma tissues and normal brain samples. Our results revealed a significant increase in FAM3C expression across different grades of glioma compared to normal brain tissues, with expression levels positively correlated with the pathological WHO grading of gliomas (Figure 1D,E). Furthermore, qRT‐PCR analysis conducted on glioma cell lines demonstrated elevated FAM3C expression levels relative to normal brain cell lines, with the highest expression observed in the U87MG cell line (Figure 1F). Survival prognosis analysis based on GEPIA and CGGA data consistently indicated that high FAM3C expression correlated with a shorter survival time in glioma patients (Figure 1G–I). Cox regression analysis performed on mRNA expression profile and clinical data from 693 patients sourced from CGGA confirmed the independent prognostic significance of FAM3C expression. Notably, high mRNA expression of FAM3C was found to be independently associated with significantly shorter overall survival in glioma patients, even after accounting for clinical variables such as World Health Organization classification, tumor type, chemotherapy, and 1p19q status (Table 2). Thus, the expression level of FAM3C emerges as a robust independent prognostic factor for glioma patients.

**TABLE 2:** Univariate and multivariate analyses of prognostic parameters in the Chinese Glioma Genome Atlas (CGGA) database overall survival (OS).

Variable	Univariate analysis		Multivariate analysis	
	HR (95% CI)	*p*	HR (95% CI)	*p*
FAM3C expression	1.012 (1.008–1.017)	< 0.001	1.006 (1.000–1.011)	0.047
WHO grade				
II				
III	0.112 (0.076–0.167)	< 0.001	0.159 (0.099–0.254)	< 0.001
IV	0.393 (0.282–0.548)	< 0.001	0.494 (0.346–0.704)	< 0.001
Age	1.920 (1.466–2.514)	< 0.001		
Tumor type	2.874 (2.160–3.824)	< 0.001	2.981 (2.105–4.222)	< 0.001
Radiotherapy	0.632 (0.457–0.872)	0.005		
Chemotherapy	1.445 (1.078–1.937)	0.014	0.590 (0.422–0.825)	0.002
IDH status	2.820 (2.136–3.723)	< 0.001		
1p/19q Codel	0.170 (0.104–0.277)	< 0.001	0.269 (0.158–0.458)	< 0.001

### 3.2 Knockdown of FAM3C Inhibits the Malignant Phenotype of Gliomas

**FIGURE 2:**
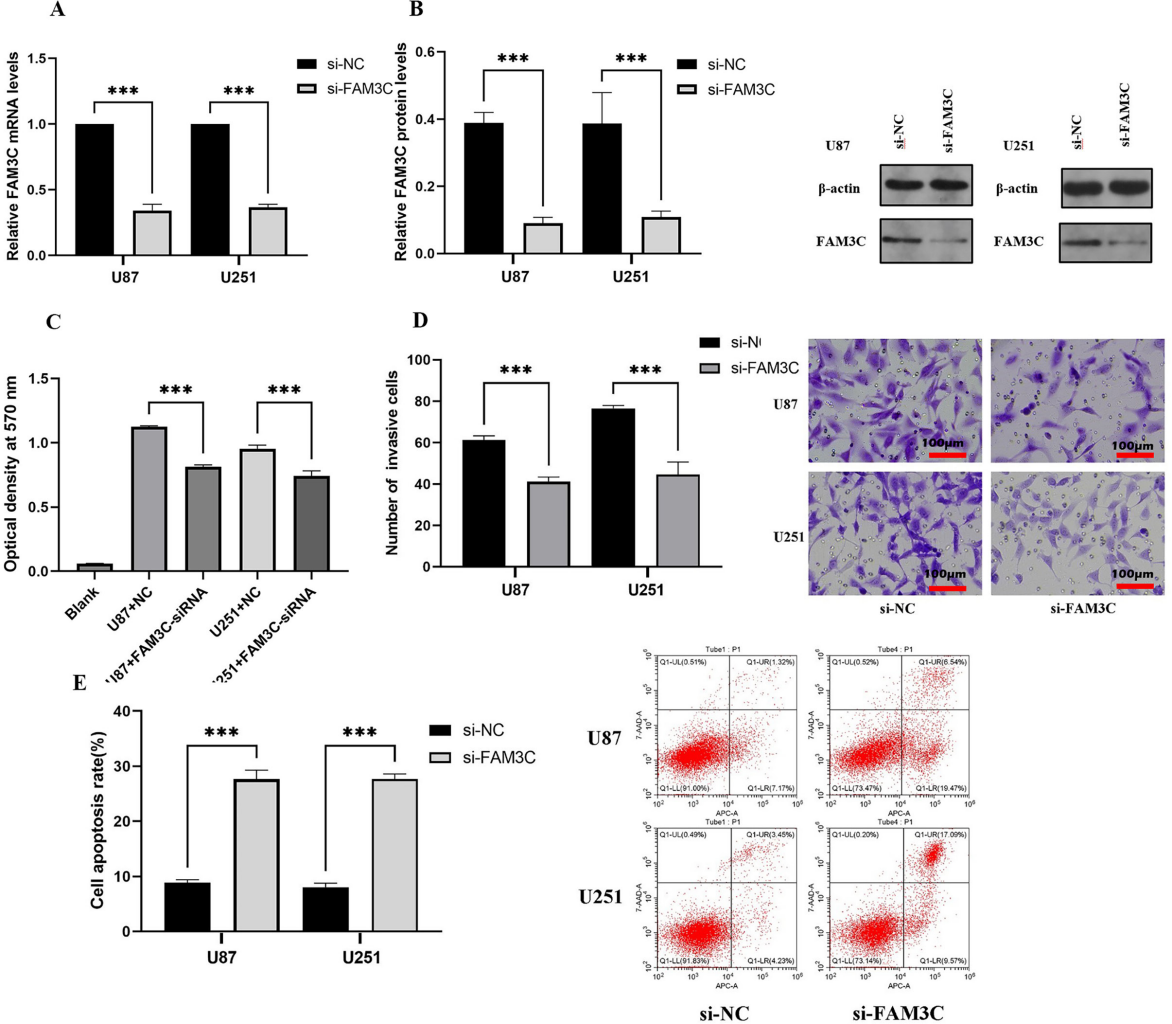
Knockdown of FAM3C inhibits the malignant phenotype of gliomas. (A, B) RT‐qPCR and western blot were used to detect the expression of FAM3C. (C) Influence of FAM3C on the proliferation of U251 and U87 glioma cells was detected by MTT. (D) Effects of FAM3C on the invasion ability of U251 and U87 glioma cells were detected by Transwell. (E) Effect of FAM3C on apoptosis level in U87 and U251 glioma cells was analyzed by flow cytometry assay. ***p < 0.001.

We proceeded to investigate the biological functions of FAM3C in gliomas. First, we employed RNA interference to knock down FAM3C expression in both the U87 and U251 cell lines, confirming the efficacy of knockdown through qRT‐PCR and western blotting analyses (Figure 2A,B). Subsequently, to assess the impact of FAM3C on the malignant phenotype of gliomas, we conducted a series of functional assays. Specifically, we used the MTT assay to evaluate proliferative ability (Figure 2C), the Transwell assay to assess invasive potential (Figure 2D), and flow cytometry to measure apoptotic ability (Figure 2E). Notably, the knockdown of FAM3C resulted in significant inhibition of proliferation, invasion, and enhanced apoptosis in the U87 cell line. Consistently, similar observations were made in U251 cells, further underscoring the role of FAM3C in promoting these malignant phenotypes in glioma cells. In summary, our findings demonstrated that FAM3C knockdown effectively restrains the proliferation and invasion and augments the apoptotic capabilities of glioma cells.

### 3.3 FAM3C Overexpression Promotes a Malignant Phenotype in Gliomas

**FIGURE 3:**
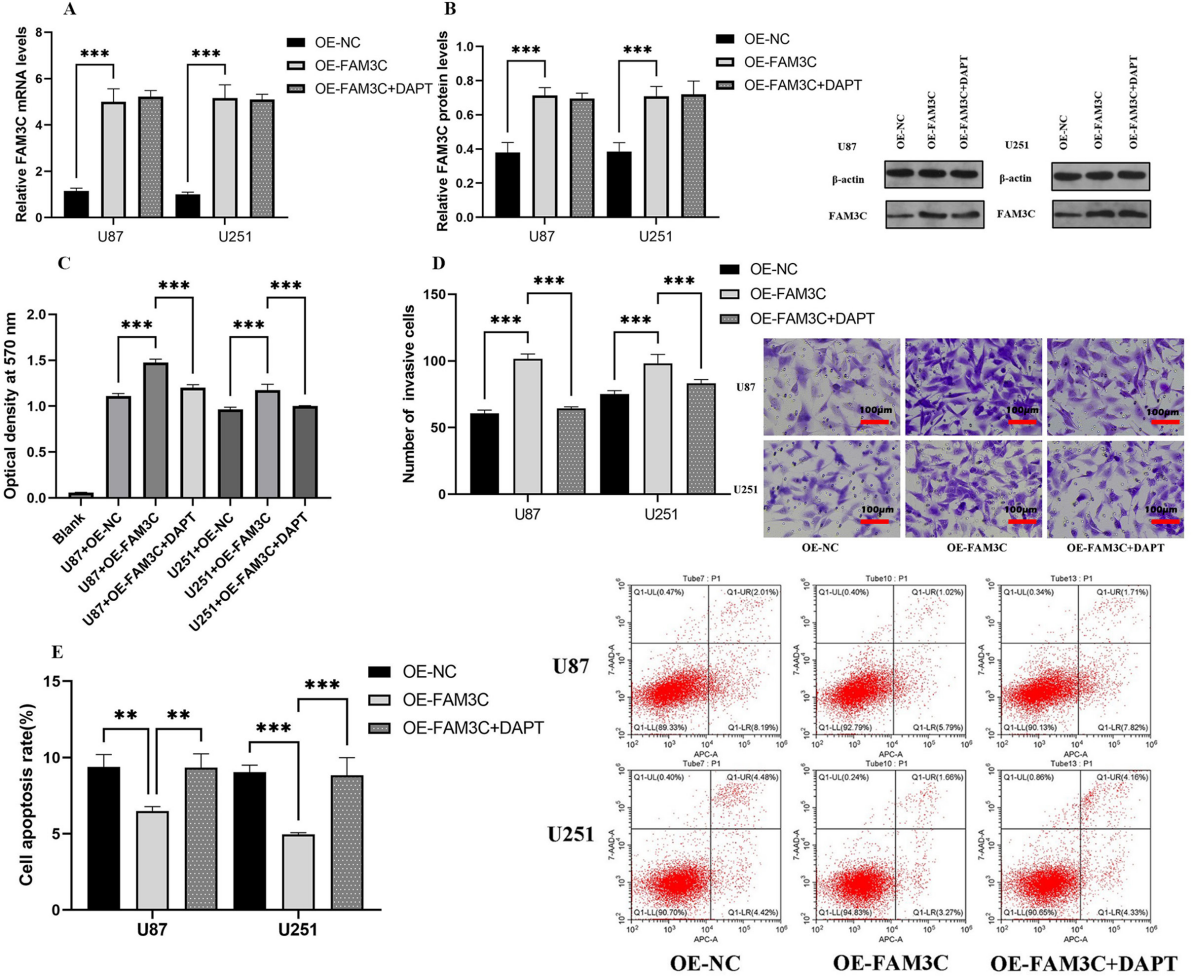
FAM3C overexpression promotes a malignant phenotype in gliomas. (A, B) RT‐qPCR and western blot were used to detect the expression of FAM3C. (C) Influence of FAM3C on the proliferation of U251 and U87 glioma cells was detected by MTT. (D) Effects of FAM3C on the invasion ability of U251 and U87 glioma cells were detected by Transwell. (E) Effect of FAM3C on the apoptosis level in U87 and U251 glioma cells was analyzed by flow cytometry assay. **p < 0.01; ***p < 0.001.

In order to delve deeper into the biological role of FAM3C in glioma cells, we overexpressed FAM3C in both the U87 and U251 cell lines, confirming the efficiency of overexpression through qRT‐PCR and western blotting (Figure 3A,B). Subsequently, to assess the impact of FAM3C on malignant phenotypes of gliomas, we performed a battery of functional assays. Specifically, we utilized the MTT assay to assess the proliferative capacity (Figure 3C), the Transwell assay to evaluate the invasive capacity (Figure 3D), and flow cytometry to measure the apoptotic capacity (Figure 3E). Remarkably, overexpression of FAM3C markedly enhanced the proliferative and invasive abilities and inhibited the apoptotic abilities of the U87 cell line. Similarly, consistent results were observed in U251 cells, further highlighting the role of FAM3C in promoting this malignant phenotype in glioma cells. In essence, our findings suggest that the overexpression of FAM3C significantly augments the proliferative and invasive capacities and decreases the apoptotic capacities of glioma cells.

### 3.4 FAM3C Promotes EMT and Activates Notch Signaling Pathway in Glioma Cells In Vitro

**FIGURE 4:**
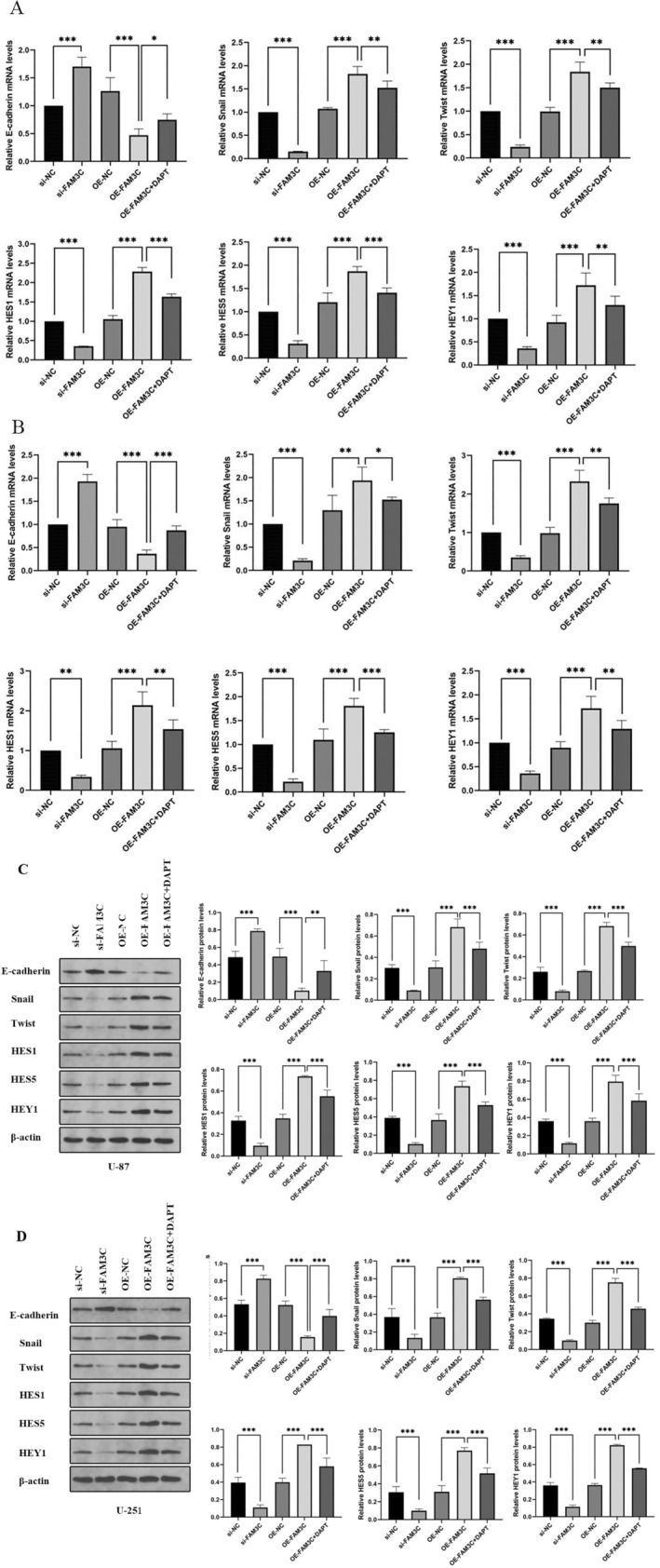
FAM3C promoted the EMT‐like process and activated the Notch signaling pathway in glioma cells in vitro. (A, B) RT‐qPCR was used to detect the expression of E‐cadherin, Snail, Twist, HES1, HES5, and HEY1 after control and treated groups in U87NG and U251. (C, D) Western blot was used to detect the expression of E‐cadherin, Snail, Twist, HES1, HES5, and HEY1 after control and treated groups in U87NG and U251. *p < 0.05; **p < 0.01; ***p < 0.001.

QRT‐PCR and western blotting experiments were performed in both U87 and U251 cells. Knockdown of FAM3C led to a significant increase in the epithelial‐associated marker E‐cadherin, along with a decrease in mesenchymalization‐related markers Snail and Twist, as well as diminished expression of Notch signaling markers HES1, HES5, and HEY1, evident at both RNA and protein levels. Conversely, overexpression of FAM3C elicited contrasting effects compared to the knockdown group. Moreover, when the Notch pathway blocker DAPT was introduced to both the U87 and U251 cell lines overexpressing FAM3C, it resulted in increased E‐cadherin levels and reduced expression of Snail, Twist, HES1, HES5, and HEY1 compared to the FAM3C overexpression group (Figure 4A–D). In functional assays including MTT, Transwell, and flow cytometry apoptosis assays, comparison between the overexpression group with DAPT addition and the sole FAM3C overexpression group revealed decreased proliferation, reduced invasive ability, and increased apoptotic cell death in both U87 and U251 cell lines (Figure 3C–E). Collectively, these data suggest FAM3C in driving epithelial mesenchymal transition in gliomas through the modulation of the Notch signaling pathway.

### 3.5 In Vivo Experiments Reaffirm that FAM3C Affects Glioma Cell Proliferation and Epithelial Mesenchymal Transition by Mediating the Notch Signaling Pathway

**FIGURE 5:**
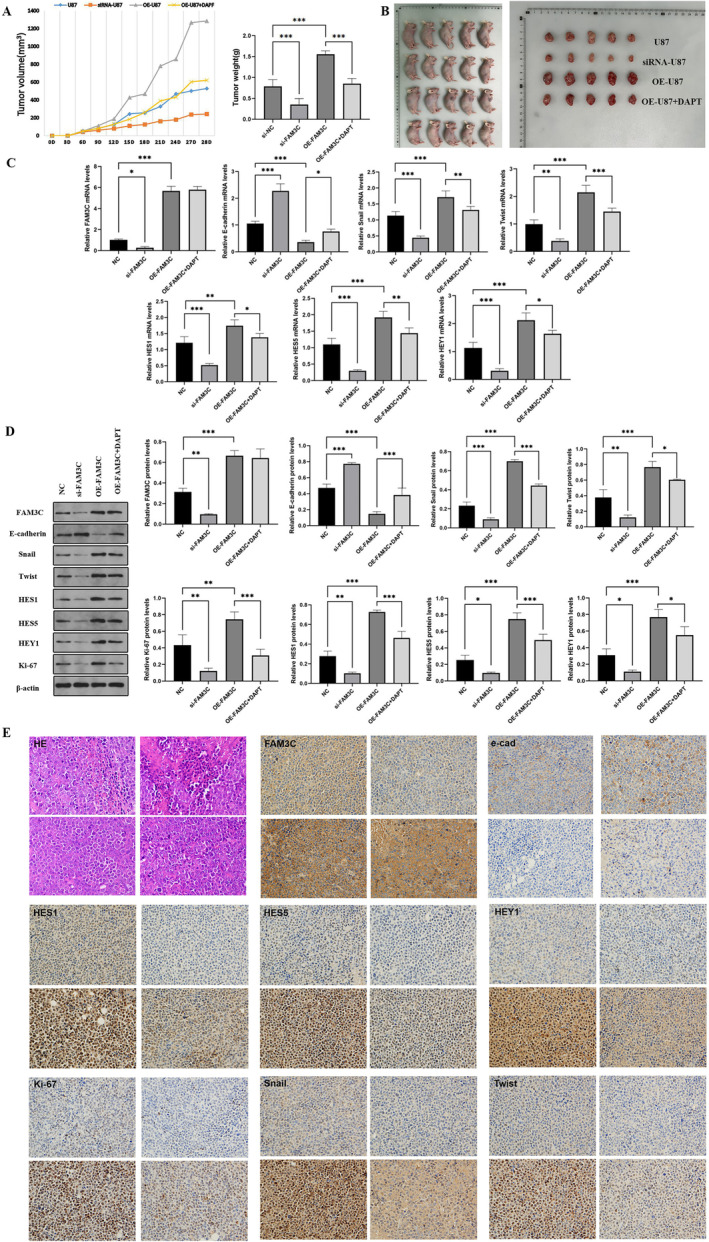
FAM3C promoted the EMT‐like process and activated the Notch signaling pathway in U87 in vivo. (A, B) U87, shRNA‐U87, oe‐U87, and oe‐U87 + DAPF cell lines were injected into rats subcutaneously and cultured for 28 days to observe the changes of tumor volume, weight, and size. (C, D) RT‐qPCR and western blot were used to detect the expression of FAM3C, E‐cadherin, Snail, Twist, Ki‐67, HES1, HES5, and HEY1. (E) Representative images of the subcutaneous xenograft tumor H&E staining of U87 and detection of FAM3C, E‐cadherin, Snail, Twist, Ki‐67, HES1, HES5, and HEY1 by IHC. *p < 0.05; **p < 0.01; *** *p* < 0.001.

We further evaluated the impact of FAM3C on U87 cell proliferation and EMT using a xenograft model. U87 cell lines, including siRNA‐U87, OE‐U87, OE‐U87, and OE‐U87 + DAPT, were subcutaneously injected into mice and allowed to grow for 28 days. Consistent with our previous findings, the average tumor volume in the knockdown group was significantly reduced compared to the control group, while the overexpression group exhibited an increased tumor volume. Notably, the addition of Notch pathway blocker results in a smaller tumor size compared to the overexpression group alone (Figure 5A,B). Tumor specimens were subjected to histomorphological analysis and quantitative analysis. Through RT‐qPCR, western blotting (Figure 5C,D), HE staining, and immunohistochemistry (Figure 5E), we observed that knockdown of FAM3C led to decreased expression of Snail, Twist, HES1, HES5, HEY1, and Ki67, alongside an elevation in the E‐cadherin level. Conversely, overexpression of FAM3C resulted in the opposite result. Notably, when FAM3C overexpression was combined with DAPT treatment, the tumor size was reduced compared to the overexpression group alone. Furthermore, this combination led to a decreased expression of Snail, Twist, HES1, HES5, HEY1, and Ki67, while E‐cadherin expression was elevated compared to the overexpression group. In summary, these in vivo findings further indicate the role of FAM3C‐mediated Notch signaling pathway in glioma proliferation and epithelial mesenchymal transition.

## 4 Discussion

FAM3C, belonging to the FAM3 family, exhibits regulatory functions in lipid and glucose metabolism, osteogenic differentiation, and retinal laminar flow formation. Recent studies have unveiled its close association with the pathogenesis of various cancers [10, 11]. Thuya et al. identified FAM3C overexpression in tumor‐derived extracellular vesicles (TDE) in non‐small‐cell lung cancer, facilitating cell invasion and distant metastasis [8]. Moreover, Wang et al. observed that tumor‐associated neutrophils regulated significant FAM3C production in gastric cancer cells via TGFβ1, thereby mediating tumor cell EMT. This cascade fueled tumor aggressiveness, culminating in the formation of lymphoid tumor cell–neutrophil clusters and subsequent tumor lymph node metastasis [9]. Additionally, Jin et al. demonstrated that FAM3C inhibited the proliferation and invasion of esophageal cancer under miR‐574‐3p‐mediated regulation, suggesting its potential as a therapeutic target in esophageal cancer treatment [7]. To investigate the role of FAM3C in glioma, our study initially assessed its expression level and prognostic implications using online databases. Subsequently, we analyzed glioma tissues obtained from our patients, along with cultured glioma cell lines and intracranial tumors from xenograft mouse models at the RNA and protein levels through PCR and western blotting experiments, respectively. Both in vivo and in vitro analyses revealed a significant upregulation of FAM3C expression in gliomas.

EMT has been recognized as a dynamic developmental process wherein epithelial cells undergo a reversible transformation into mesenchymal cells, acquiring corresponding mesenchymal functionalities. This process is activated in various pathological contexts, such as chronic inflammation, fibrosis, and wound healing [12, 13, 14]. Within the scope of long‐term tumor studies, EMT stands as one of the pivotal contributors to tumor progression, invasive migration, and drug resistance development [15, 16, 17, 18]. In order to study the relationship between FAM3C and glioma‐associated EMT, we conducted knockdown and overexpressed experiments targeting FAM3C in U87 and U251 cells. Our findings indicated a reduction in tumor cell proliferation, invasion, and apoptosis following FAM3C knockdown, accompanied by an elevation in E‐cadherin level and a decrease in Snail and Twist expression in both in vivo and in vitro settings, whereas FAM3C overexpression yielded contrasting outcomes. Thus, it is evident that elevated FAM3C expression fosters the proliferation, invasion, apoptosis, and EMT of glioma cells.

The Notch signaling pathway, known for its high conservation across species, plays a crucial role in various physiological processes, including normal cell proliferation, differentiation, apoptosis, embryonic development, and tissue growth homeostasis. Additionally, the involvement of this pathway in tumorigenesis has been extensively documented [19, 20, 21]. Reports increasingly highlight the multifaceted contributions of the Notch pathway in glioma pathogenesis, including the regulation of glioma stem cell proliferation and differentiation, facilitation of a hypoxic tumor microenvironment, promotion of neovascularization, enhancement of chemo‐ as well as radio‐resistance, and mediation of mesenchymal transition in tumor epithelial cells, all of which drive glioma development, recurrence, and metastasis [22, 23, 24, 25, 26]. To elucidate the interplay between FAM3C and the Notch signaling pathway, we supplemented FAM3C‐overexpressing cell lines with DAPT, a Notch signaling pathway inhibitor, and compared them with control‐overexpressing cell lines. Our results revealed that tumor cell proliferation and invasion were significantly reduced, and apoptosis was enhanced in the FAM3C‐overexpressing group compared to the control‐overexpressing group,alongside elevated E‐cadherin levels both in in vivo and in vitro experiments and decreased expression of Snail, Twist, HES1, HES5, and HEY1. These findings underscore the notion that FAM3C suppression mediates the Notch signaling pathway, consequently inhibiting glioma proliferation, invasion, epithelial mesenchymal transition, and promotion of apoptosis. However, some studies have indicated that in patients with Alzheimer's disease, Notch signaling and γ‐secretase activity are not influenced by FAM3C, which is inconsistent with the findings of our study [27]. Previous research has demonstrated that the activation of the Notch pathway requires the action of three different secretases, with three successive cleavage events, of which γ‐secretase is only a part. Furthermore, Notch pathway activation can occur through classical pathways or through nonclassical pathways that do not depend on the Notch intracellular domain (NICD) [28]. Therefore, further exploration of the relationship between the Notch pathway and FAM3C will be one of the directions for our future research.

FAM3C exhibits a variety of functions across different disease types, suggesting that it may operate through distinct signaling pathways in various cancers. However, the role of FAM3C in GBM needs to be more understood. Given its involvement in multiple cancers, relying solely on FAM3C as a prognostic marker for GBM may lack specificity, as its functional mechanisms in other cancers could interfere. The diversity of its pathway mechanisms could also limit its efficacy as a GBM therapeutic target, particularly in selecting treatments that may not address all relevant pathways. Moreover, most current studies on FAM3C are still at the basic research or preclinical stages, lacking the need for large‐scale clinical data specific to GBM. While findings from other cancers provide some justification for its use as a biomarker, the biological characteristics of each cancer differ. Therefore, the prognostic and therapeutic potential of FAM3C in GBM must be validated through large‐scale clinical trials. The multifunctionality and variability in FAM3C mechanisms across different cancers might limit its application as a specific biomarker for GBM, and its broad mechanistic role as a therapeutic target could pose unpredictable side effects. In future research, we focus on developing FAM3C inhibitors, combination therapies with Notch pathway inhibitors, inhibition of FAM3C‐mediated EMT, interventions targeting treatment resistance, and potential combination strategies with other therapies. These efforts will explore the prospective applications of FAM3C in GBM treatment.

## 5 Conclusions

Our study reveals that silencing FAM3C gene mediates the Notch pathway, impacting glioma cell proliferation, invasion, apoptosis, and epithelial mesenchymal transition. Hence, FAM3C may be chosen as a potential biomarker for clinical diagnosis and a promising therapeutic target in glioma treatment.

## Author Contributions

**Xiaochao Xia:** resources (lead), software (lead), validation (lead), writing – original draft (lead). **Zihao Wang:** validation (equal), visualization (equal). **Lvmeng Song:** resources (equal), software (equal), validation (equal). **Yinchuan Cheng:** resources (equal), validation (equal). **Ping Xiong:** software (equal), supervision (equal), validation (equal). **Shun Li:** supervision (equal), writing – review and editing (equal).

## Ethics Statement

The use of human tissues was approved by the ethics committee of Affiliated Hospital of North Sichuan Medical College. All animal studies were approved by the Institutional Animal Care and Use Committee of North Sichuan Medical College.

## Conflicts of Interest

The authors declare no conflicts of interest.

## Data Availability

All data generated or analyzed during this study are included in this article. Further inquiries can be directed to the corresponding author.

## References

[1] B. Kristensen , L. Priesterbach‐Ackley , J. Petersen , and P. Wesseling , “Molecular Pathology of Tumors of the Central Nervous System,” Annals of Oncology 30, no. 8 (2019): 1265–1278. 31124566 10.1093/annonc/mdz164 PMC6683853

[2] K. Yang , Z. Wu , H. Zhang , et al., “Glioma Targeted Therapy: Insight Into Future of Molecular Approaches,” Molecular Cancer 21, no. 1 (2022): 1–32. 35135556 10.1186/s12943-022-01513-z PMC8822752

[3] B. Huang , Z. Yu , and R. Liang , “Effect of Long‐Term Adjuvant Temozolomide Chemotherapy on Primary Glioblastoma Patient Survival,” BMC Neurology 21 (2021): 1–8. 34724914 10.1186/s12883-021-02461-9 PMC8561964

[4] Y. Zhu , Z. Pu , G. Wang , Y. Wang , N. Li , and F. Peng , “FAM3C: An Emerging Biomarker and Potential Therapeutic Target for Cancer,” Biomarkers in Medicine 15, no. 5 (2021): 373–384. 33666514 10.2217/bmm-2020-0179

[5] Z. H. Gao , C. Lu , Z. N. Wang , et al., “ILEI: A Novel Marker for Epithelial‐Mesenchymal Transition and Poor Prognosis in Colorectal Cancer,” Histopathology 65, no. 4 (2014): 527–538. 24738665 10.1111/his.12435

[6] U. Schmidt , G. Heller , G. Timelthaler , et al., “The FAM3C Locus That Encodes Interleukin‐Like EMT Inducer (ILEI) is Frequently Co‐Amplified in MET‐Amplified Cancers and Contributes to Invasiveness,” Journal of Experimental & Clinical Cancer Research 40, no. 1 (2021): 69. 33596971 10.1186/s13046-021-01862-5 PMC7890988

[7] L. L. Jin , S. J. Zhang , G. X. Lu , F. Lv , R. Shang , and J. Yang , “miR‐574‐3p Inhibits Proliferation and Invasion in Esophageal Cancer by Targeting FAM3C and MAPK1,” Kaohsiung Journal of Medical Sciences 36, no. 5 (2020): 318–327. 31880039 10.1002/kjm2.12176 PMC11896219

[8] W. L. Thuya , L. R. Kong , N. L. Syn , et al., “FAM3C in Circulating Tumor‐Derived Extracellular Vesicles Promotes Non‐small Cell Lung Cancer Growth in Secondary Sites,” Theranostics 13, no. 2 (2023): 621. 36632230 10.7150/thno.72297 PMC9830426

[9] Y. Wang , X. Li , T. Zhang , et al., “Neutrophils Promote Tumor Invasion via FAM3C‐Mediated Epithelial‐To‐Mesenchymal Transition in Gastric Cancer,” International Journal of Biological Sciences 19, no. 5 (2023): 1352–1368. 37056931 10.7150/ijbs.79022 PMC10086748

[10] A. Mura , “Molecular Mechanisms of Fam3c Cardioprotective Activity,” 2023, 10.15168/11572_371430.

[11] A. N. Woosley , A. C. Dalton , G. S. Hussey , et al., “TGFβ Promotes Breast Cancer Stem Cell Self‐Renewal Through an ILEI/LIFR Signaling Axis,” Oncogene 38, no. 20 (2019): 3794–3811. 30692635 10.1038/s41388-019-0703-z PMC6525020

[12] Z. Huang , Z. Zhang , C. Zhou , L. Liu , and C. Huang , “Epithelial–Mesenchymal Transition: The History, Regulatory Mechanism, and Cancer Therapeutic Opportunities,” MedComm 3, no. 2 (2022): e144. 35601657 10.1002/mco2.144 PMC9115588

[13] C. Yu , Q. Liu , C. Chen , J. Yu , and J. Wang , “Landscape Perspectives of Tumor, EMT, and Development,” Physical Biology 16, no. 5 (2019): 051003. 31067516 10.1088/1478-3975/ab2029

[14] S. Brabletz , H. Schuhwerk , T. Brabletz , and M. P. Stemmler , “Dynamic EMT: A Multi‐Tool for Tumor Progression,” EMBO Journal 40, no. 18 (2021): e108647. 34459003 10.15252/embj.2021108647 PMC8441439

[15] G. Pan , Y. Liu , L. Shang , F. Zhou , and S. Yang , “EMT‐Associated microRNAs and Their Roles in Cancer Stemness and Drug Resistance,” Cancer Communications 41, no. 3 (2021): 199–217. 33506604 10.1002/cac2.12138 PMC7968884

[16] M. Hashemi , H. Z. Arani , S. Orouei , et al., “EMT Mechanism in Breast Cancer Metastasis and Drug Resistance: Revisiting Molecular Interactions and Biological Functions,” Biomedicine & Pharmacotherapy 155 (2022): 113774. 36271556 10.1016/j.biopha.2022.113774

[17] S. Papanikolaou , A. Vourda , S. Syggelos , and K. Gyftopoulos , “Cell Plasticity and Prostate Cancer: The Role of Epithelial–Mesenchymal Transition in Tumor Progression, Invasion, Metastasis and Cancer Therapy Resistance,” Cancers 13, no. 11 (2021): 2795. 34199763 10.3390/cancers13112795 PMC8199975

[18] D. Greaves and Y. Calle , “Epithelial Mesenchymal Transition (EMT) and Associated Invasive Adhesions in Solid and Haematological Tumours,” Cells 11, no. 4 (2022): 649. 35203300 10.3390/cells11040649 PMC8869945

[19] V. Kumar , M. Vashishta , L. Kong , et al., “The Role of Notch, Hedgehog, and Wnt Signaling Pathways in the Resistance of Tumors to Anticancer Therapies,” Frontiers in Cell and Developmental Biology 9 (2021): 650772. 33968932 10.3389/fcell.2021.650772 PMC8100510

[20] J. O. Misiorek , A. Przybyszewska‐Podstawka , J. Kałafut , et al., “Context Matters: NOTCH Signatures and Pathway in Cancer Progression and Metastasis,” Cells 10, no. 1 (2021): 94. 33430387 10.3390/cells10010094 PMC7827494

[21] A. Akil , A. K. Gutiérrez‐García , R. Guenter , et al., “Notch Signaling in Vascular Endothelial Cells, Angiogenesis, and Tumor Progression: An Update and Prospective,” Frontiers in Cell and Developmental Biology 9 (2021): 177. 10.3389/fcell.2021.642352 PMC7928398 33681228

[22] Y. Wang , Y. Cheng , Q. Yang , L. Kuang , and G. Liu , “Overexpression of FOXD2‐AS1 Enhances Proliferation and Impairs Differentiation of Glioma Stem Cells by Activating the NOTCH Pathway via TAF‐1,” Journal of Cellular and Molecular Medicine 26, no. 9 (2022): 2620–2632. 35419917 10.1111/jcmm.17268 PMC9077300

[23] J.‐S. Ren , W. Bai , J.‐J. Ding , et al., “Hypoxia‐Induced AFAP1L1 Regulates Pathological Neovascularization via the YAP‐DLL4‐NOTCH Axis,” Journal of Translational Medicine 21, no. 1 (2023): 651. 37737201 10.1186/s12967-023-04503-x PMC10515434

[24] M. Below and C. Osipo , “Notch Signaling in Breast Cancer: A Role in Drug Resistance,” Cells 9, no. 10 (2020): 2204. 33003540 10.3390/cells9102204 PMC7601482

[25] A. Nandi , R. Debnath , A. Nayak , et al., “Dll1‐Mediated Notch Signaling Drives Tumor Cell Cross‐Talk With Cancer‐Associated Fibroblasts to Promote Radioresistance in Breast Cancer,” Cancer Research 82, no. 20 (2022): 3718–3733. 36007109 10.1158/0008-5472.CAN-21-1225 PMC10204098

[26] R. Farahzadi , B. Valipour , E. Fathi , et al., “Oxidative Stress Regulation and Related Metabolic Pathways in Epithelial–Mesenchymal Transition of Breast Cancer Stem Cells,” Stem Cell Research & Therapy 14, no. 1 (2023): 1–19. 38017510 10.1186/s13287-023-03571-6 PMC10685711

[27] H. Hasegawa , L. Liu , I. Tooyama , et al., “The FAM3 Superfamily Member ILEI Ameliorates Alzheimer's Disease‐Like Pathology by Destabilizing the Penultimate Amyloid‐β Precursor,” Nature Communications 5 (2014): 3917. 10.1038/ncomms4917 24894631

[28] D. Sprinzak and S. C. Blacklow , “Biophysics of Notch Signaling,” Annual Review of Biophysics 50 (2021): 157–189. 10.1146/annurev-biophys-101920-082204 PMC8105286 33534608

